# How Group Size Affects Vigilance Dynamics and Time Allocation Patterns: The Key Role of Imitation and Tempo

**DOI:** 10.1371/journal.pone.0018631

**Published:** 2011-04-15

**Authors:** Pablo Michelena, Jean-Louis Deneubourg

**Affiliations:** Unit of Social Ecology, Université libre de Bruxelles (ULB), Brussels, Belgium; University of Zürich, Switzerland

## Abstract

In the context of social foraging, predator detection has been the subject of numerous studies, which acknowledge the adaptive response of the individual to the trade-off between feeding and vigilance. Typically, animals gain energy by increasing their feeding time and decreasing their vigilance effort with increasing group size, without increasing their risk of predation (‘group size effect’). Research on the biological utility of vigilance has prevailed over considerations of the mechanistic rules that link individual decisions to group behavior. With sheep as a model species, we identified how the behaviors of conspecifics affect the individual decisions to switch activity. We highlight a simple mechanism whereby the group size effect on collective vigilance dynamics is shaped by two key features: the magnitude of social amplification and intrinsic differences between foraging and scanning bout durations. Our results highlight a positive correlation between the duration of scanning and foraging bouts at the level of the group. This finding reveals the existence of groups with high and low rates of transition between activies, suggesting individual variations in the transition rate, or ‘tempo’. We present a mathematical model based on behavioral rules derived from experiments. Our theoretical predictions show that the system is robust in respect to variations in the propensity to imitate scanning and foraging, yet flexible in respect to differences in the duration of activity bouts. The model shows how individual decisions contribute to collective behavior patterns and how the group, in turn, facilitates individual-level adaptive responses.

## Introduction

Aggregation and cooperative interactions among animals often result in collective behaviors that benefit group members [1]. However our understanding of exactly how individual interactions scale to collective properties, and the consequences of this process for individual survival, is limited. In the context of social foraging, collective vigilance and predator detection have been the subject of numerous models and quantitative studies. These studies acknowledge 1) the adaptive response of the individual to the trade-off between feeding and vigilance by which they gain energy and decrease their vigilance in response to increasing group size and 2) the fact that the probability of detecting an approaching predator is greater in larger groups, because the probability that at least one animal will be vigilant at any time increases with group size (the ‘group size effect’, [2]–[3]). While the first prediction of such a group size effect is supported by empirical evidence in various taxa, the second prediction is sometimes empirically supported [4]–[5], sometimes not [6]. In other cases, the collective vigilance increases up to a pivotal group size and then decreases [7]. Until now, consideration of the biological utility of vigilance, from an ecological standpoint, has prevailed over consideration of the organizing principles and most of these studies failed to explore the mechanistic rules involved. Consequently, the true nature of the link between individual decisions and group behavior remains elusive.

A growing interest in the principles that shape collective decisions has drawn attention to the key components of individual coordination rules [8]–[9]. Basically, an individual within a group responds to both internal and external stimuli, and this is generally formalized by the probability of spontaneously switching between activities, which is modulated by the presence or activity of conspecifics [10]. In most cases, the non-linearity in facilitated responses to others results in activity coordination at the group level [11]–[12]. In an early individual-based model of collective vigilance, Bahr and Bekoff [13] assumed a certain level of vigilance coordination in the group, where each individual tended to perform the ‘opposite’ behavior to its immediate neighbors (coordinated vigilance). This rule however, did not receive experimental support [14]. Instead, many studies show that scanning and feeding bouts are often synchronized within the group, therefore rejecting the hypothesis of coordinated vigilance (birds [15]–[17], ungulates [5]–[6], rodents [4] and marsupials [7]).

The aim of the present study is to characterize the decision-making rules that underlie collective vigilance and group size effects on foraging and scanning behavior in social herbivores. Using an experimental and a theoretical approach, we develop a general model of time allocation patterns in group-living animal species prone to imitative behavior. Since the main difficulty of interpreting vigilance data is the number of confounding variables affecting vigilance, e.g. predation risk, food resource variation, social relationships, individual variation and group size and composition [18]–[20], we designed experiments in which we standardized conditions in terms of sex, age, and familiarity between individuals, as well as animal density and pasture conditions. Among large herbivores, vigilance behaviour was mainly studied in ungulate species. Few of those studies however were carried out under experimental conditions because of the difficulties to keep wild ungulate species under controlled conditions. Despite domestication, sheep adopt similar behaviours to those of their wild counterparts [21]–[22]. Recent studies showed that sheep constitute a good biological model to address questions about collective dynamics in herbivore species and to assess generic rules that govern individual decision-making processes [23]–[26]. In the present paper, the model, using parameter values derived from experimental data, provides a formal link between individual and collective behavior. With the model, we explore how key factors acting at the level of individual decision-making affect the dynamics of collective vigilance.

## Materials and Methods

### Ethics Statement

Animal care and experimental manipulations were in accordance with the rules of the French committee of animal experimentation ethics. Institutional approval was not necessary for such a study since the need for animal care during experiments was not different from normal farm management conditions.

### Study area and subjects

Fieldwork was carried out at the experimental farm of Domaine du Merle (5.74°E and 48.50°N) in the South of France, from November 2003 to February 2004. We used 34 Arles merino horned males (median age = 3 years, range = 1–7) randomly chosen from a flock of males (*n* = 66). The subjects were familiarized with one another by being kept together on a 1-ha pasture for 5 weeks before starting the experiments. All animals were identified with a number on both flanks and on the rump.

### Experimental set-up and procedure

A 25 m-diameter arena, placed at a distance of 22.5 m from a 7 m-high tower, was established using sheep fences in a field of native wet Crau meadows, predominantly composed of graminoids, clover *Trifolium* sp. and plantain *Plantago lanceolata* [27]. Visual contact with the immediate surroundings was prevented by a 1.2 m-high green polypropylene net.

The experimental design consisted of testing a series of groups of 2, 4, 6 or 8 individuals in this arena. The order in which group sizes were tested was chosen randomly and 5 replications were conducted for each group size. 20 of the 34 individuals were randomly allocated to one group size in each replicate. Familiarization of individuals with the experimental set-up and their social group began at 10:00 the day before experiments, by introducing the group to be tested into a waiting area. At 17:00, the group was introduced into the experimental arena, in preparation for testing the next day.

The tower and the arena were moved 3 times within the field, in order to prevent depletion of the pasture. Sward heights were measured (±0.5 cm) within the arenas, using an HFRO sward stick, the evening before the groups were introduced. Sward height and estimated herbage biomass did not vary significantly for the different group sizes. Full details of the arenas and experimental procedures can be found in Michelena et al. [23].

### Data collection

The behavior of sheep in each arena was recorded on video from 10:00 to 16:00 with a digital camcorder (Sony DCR-TRV950 E) mounted on the top of the tower and connected to a PowerBook laptop. The laptop was programmed to take a snapshot from the camera every second over a 6-hour period (*n* = 21,600 seconds).

From the digital snapshots (*n* = 432,000) collected during all the 6-hour recordings, the behavior of each individual was identified on a replay monitor and was classified as foraging, scanning or lying. In accordance with the classical behavioral repertoire of ungulates species, scanning was defined as an immobile posture with the head horizontal and raised above the column axis. Foraging activity consists in sheep grazing pasture or moving during grazing bouts [28]–[29]. Two observation days, one with a group of 8 individuals and the other one with a group of 6 individuals, were discarded from the analyses because of disturbances related to the presence of hunters near to the set-up. In addition, the behavior of some sheep was occasionally impossible to identify for short periods (*n* = 887 periods, mean duration = 95 s, range: 1–869 s) and thus considered as unknown during that time (*censored time*). All the sequences where at least one individual in the group was lying were removed from the analyses and we only considered groups where all individuals were engaged in either foraging or scanning (*n* = 238,284; mean observation time by group = 13,238 s, range: 7,927–18,090 s, no differences of the observation time between group sizes were found: *F_3,14_ = 1.05*, *P = 0.40*).

### Data Analysis

**Figure 1:**
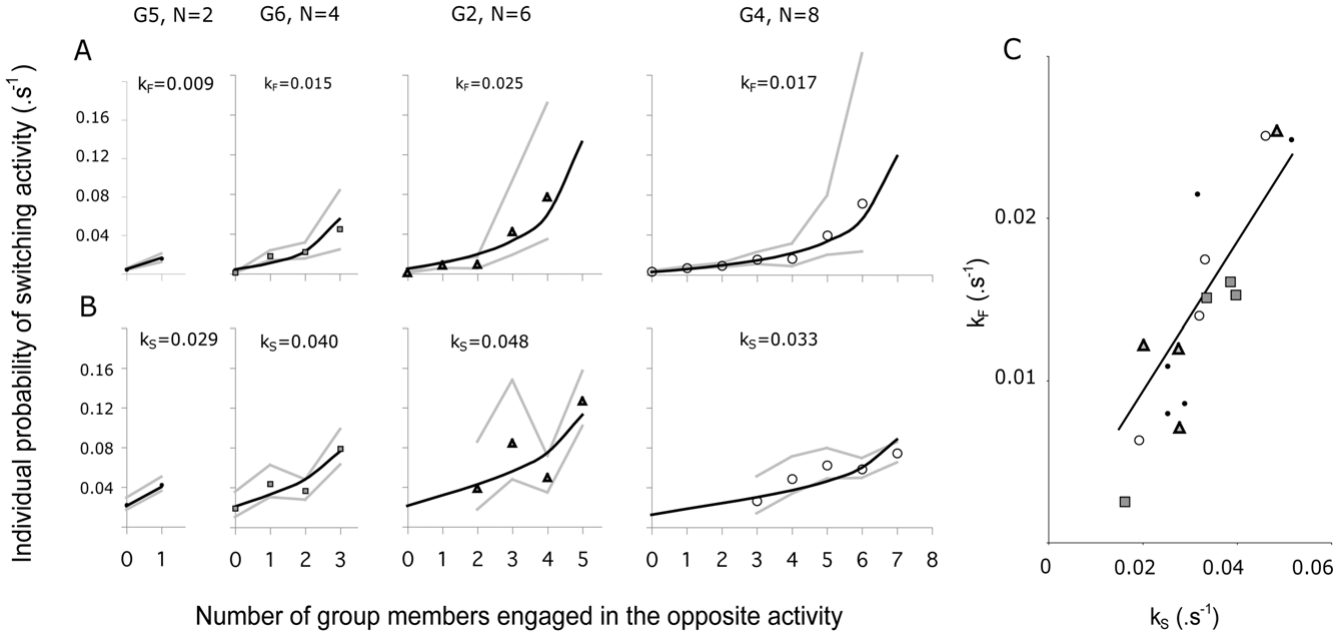
Individual probability of switching activity. Figures 1A and 1B represent the experimental (symbols: mean±CI95%) and theoretical (black lines; eq. (1)) individual probabilities of switching from foraging to scanning (*T_F→S_*) and from scanning to foraging activity (*T_S→F_*) as a function of the number of scanning or foraging conspecifics respectively. Figure 1A,B illustrate the results extracted from the analyses of 4 experimental groups composed of 2, 4, 6 and 8 individuals (G5, G6, G2 and G4, respectively). The adjusted values of the parameters k_F_ and k_S_ are indicated for each group (see eq. (1)). Fig. 1C shows the linear relationship between the k_F_ and k_S_ values for each tested group (*r^2^* = 0.80, k_F_ = (0.46)k_S_, *F*_1,17_ = 69.6, *P*<0.001). Black points, grey squares, grey triangles and open circles represent the k values of tested groups composed of 2, 4, 6 and 8 sheep, respectively.

For each group size, we identified several possible states depending on the number of individuals engaged in either scanning or foraging activities (i.e. for a group of 4 individuals, we identified 5 group states namely: 0, 1, 2, 3 or 4 individuals scanning). For each group, the lifetime of a given group state was defined as the elapsed time between the point at which any individual started scanning (or foraging) and the point at which any individual stopped scanning (or foraging). For each tested group, the lifetimes of each states were calculated using the survival package of R software and showed that the probability of switching activities was constant per unit time (i.e. a Markovian process, goodness of fit test: all P<0.05 with sample size ranging from 10 to 398 occurrences). These experimental probabilities were then fitted (i.e. Fig. 1A,B) using non-linear least squares regressions performed with SPSS (vers. 11.0, SPSS, Chicago).

## Results

### Characterization of individual decision rules: the probability of switching activity

We hypothesized that the number of conspecifics already engaged in each activity would affect the probability of an individual switching activity. In accordance with this hypothesis, for a foraging individual, the probability of scanning (*T_F→S_*) increases as the proportion of conspecifics already scanning increases (Fig. 1A). Similarly for a scanning individual, the probability of foraging (*T_S→F_*) increases as the proportion of conspecifics already foraging increases (Fig. 1B). Accordingly, the theoretical probability of switching activity corresponds to an intrinsic propensity to stop foraging (or scanning) and start scanning (or foraging) that is modulated by the ratio of stimulating and inhibiting effects exerted by the activity of the conspecifics. The experimental data were fitted with the basic function:
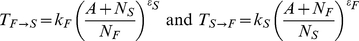
(1)


This function formulates the mimetic effect and calculates the probability of switching activity according to the activity of conspecifics. In equation (1), k_F_ and k_S_ modulate the spontaneous probabilities of starting scanning and foraging bouts respectively (i.e. corresponding to a theoretical isolated individual). N_F_ and N_S_ are the number of foraging and scanning individuals respectively. ε_S_ and ε_F_ are the coefficients of mimetic sensitivity for scanning and foraging respectively and A is a constant (see Table 1). Note that positive values of epsilons indicate mimetic behavior, while negative values indicate a propensity for coordination. For ε_S_ = ε_F_ = 0, the individual decision to switch activity is independent of the behavior of the conspecifics. In order to select the most parsimonious model, we constrained the parameter set and fitted the experimental curves with equation (1) by minimizing the residual sum of squares to estimate parameter values, assuming that the propensity to mimic the activity of others was the same for all groups, but that the spontaneous probabilities of switching activity (k_F_ and k_S_) might vary between the tested groups as a result of inter-individual differences. The best fit to the experimental data (*r^2^* = 0.86) was obtained with A = 1, ε_S_ = 0.92 and ε_F_ = 0.47, and with k_F_ and k_S_ ranging from 0.002 to 0.025 s^−1^ and 0.016 to 0.051 s^−1^ respectively (Fig. 1C). Note that for the experimentally measured value of A = 1, probabilities of switching from scanning to foraging and from foraging to scanning of a theoretical isolated sheep (or those of an individual that would not be under the social influence of conspecifics) correspond to k_S_ and k_F_ respectively. These results reveal, on the one hand, a stronger social facilitation effect for scanning than for foraging (ε_S_>ε_F_) and, on the other hand, a higher spontaneous probability of stopping scanning (k_S_>k_F_).

**Table 1:** Parameters and variables used in the model and the corresponding biological concepts.

Table of symbols	
Biological concepts in the model	
N_S_	Number of sheep scanning in the group
N_F_	Number of sheep foraging in the group
T_S→F_	Individual probability of transition from scanning to foraging activity (.s^−1^)
T_F→S_	Individual probability of transition from foraging to scanning activity (.s^−1^)
P(N_S_)	Probability of having N_S_ individual scanning in the group.
P(0)	For N_S_ equals to 0, P(0) indicates the probability that no individual in the group is scanning (all individuals are foraging).
P_coll_	Probability that at least one individual in the group is scanning, also referred as collective vigilance. Note that P_coll_ corresponds to 1-P(0).
P_indiv_	Proportion of time an individual spends scanning, and 1-Pindiv gives the proportion of time an individual spend foraging.
F0	Frequency of transition towards bouts where no individual is scanning in the group (also refered as risky bouts)
<T0>	Average duration of a risky bout
*Parameters*	
k_S_	Modulates the spontaneous (i.e. a theoretical isolated sheep) probability of an individual stopping a scanning bout to start foraging
k_F_	Modulates the spontaneous (i.e. a theoretical isolated sheep) probability of an individual stopping a foraging bout to start scanning
e_F_	Modulates the mimetic sensitivity towards the behaviour of neighbours when making the decision to start foraging
e_S_	Modulates the mimetic sensitivity towards the behaviour of neighbours when making the decision to start scanning
N	Group size
A	A constant. The fact that the experimental value of A equals to 1 in our model, makes the probability to spontaneously switching activity only depends on k_F_ and k_S_

Unexpectedly, we found a strong relationship between the fitted k_F_ and k_S_ values for each group (*r^2^* = 0.80, *F*_1,17_ = 69.6, *P*<0.001), revealing the existence of groups with high or low rate of transition in activity (referred to as ‘tempo’) (Fig. 1C). Further analyses showed that such tempos of transition of activity did not depend on group size (*F*_1,16_ = 0.0001, *P* = 0.99, see Fig. 1C). Rather, since the k_F_ and k_S_ values for a particular group correspond to the average k of the individuals within that group, these results intriguingly suggest that individuals vary in their intrinsic tempo of transition in activity.

### Implementation of individual decisions

To account for experimental fluctuations (which can be large because of a small number of individuals), we performed both stochastic simulations of the model (individual based model) and numerical resolution of differential equations (master equations), under the assumption that the state of the system, i.e. the number of individuals scanning (Ns), is described in terms of a probability function *P*(*N*_s_ = 0, 1, 2,…, N). In the individual-based model, two behavioral states are considered: scanning (S) and foraging (F). For each group, probabilities are assigned depending on the spontaneous transition rates (k_S_ and k_F_) and parameters derived from empirical data. At the beginning of a simulation run, sheep are all initialized as scanning. The probability of switching activity then depends on the number of conspecifics both in the same and in the other behavioral state (equation (1)). At each time step, the individual decision to change activity depends on a comparison between calculated probabilities and a random number between 0 and 1. These probabilities are updated at each time step. The duration of a simulation was 4 h (14,400 s), with a time step of 0.01 s/cycle. A total of 100 simulations were run for each experimental group.

The master equation describes the time evolution of the probability of the system to occupy each one of the discrete sets of states (see Supporting Information S1). *P(N_S_)* is the probability for the system to be in state *N_s_*. *N_s_* is the number of scanning individuals (*N_S_* = *0,1,….,N*). *N i*s the total number of individuals and *N_F_* is the number of foraging individuals (*N_F_* = *N*-*N_S_)*. The equation counts the processes leading the system to the sate *N_S_* and the processes removing it from this state. The evolution of the master equations (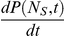) is thus given in terms of a birth and death type of master equation [30], which in reduced form reads:
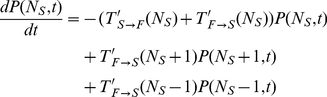
(2)


With P(N_S_, t) the probability of N_S_ individuals scanning at the time t, and T′ the probability of switching activity depending on the number of scanning (N_S_) and foraging (N_F_) individuals in a group of N individuals at the time t-1 (For further details see Supporting Information S1) .

Based on our experimental results, the master equations were fitted with the function:
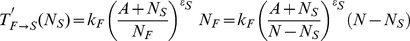
(3)and
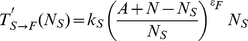
(4)


### Predictions of the model

#### Simulated vs experimental results within the range of parameter values derived from experiments

**Figure 2:**
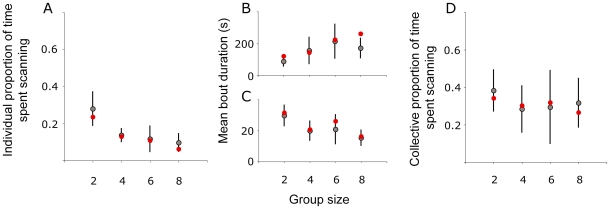
Experimental and predicted patterns of vigilance as group size increases. Figure 2 represents both the experimental (grey circles: mean±CI95%) and simulated (red circles) dynamic outcomes as a function of group size. Figure 2A shows the average proportion of time an individual spent scanning. Figure 2B and 2C represent the average duration of foraging and scanning bouts, respectively. Figure 2D shows the average proportion of time where at least one individual in the group is scanning (also referred as collective vigilance).

The simulated and experimental proportions of time devoted to scanning by an individual were similar and decreased as group size increased (Fig. 2A), as a result of both an increase in foraging bout duration (Fig. 2B) and a decline in scanning bout duration (Fig. 2C). The model also accounts for the level of collective vigilance (P_coll_) measured in experiments (Fig. 2D).

#### Theoretical generalization and model properties

With the parameters of *T_F→S_* and *T_S→F_* estimated experimentally (see Eq (1)), Eq (2) provides the overall distribution of the number of individuals scanning at any time and admits a steady-state solution (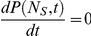), which can be computed exactly. For A = 1, it is easy to show that (see Supporting Information S2):
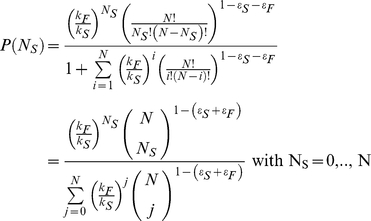
(5)


These results allow us to determine the proportion of time when at least one individual is scanning (collective vigilance: P_coll_ = 1-P(0)) and the average proportion of time devoted to scanning (P_indiv_) and foraging (1-P_indiv_) by an individual:
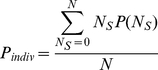
(6)


Equation (5) and (6) demonstrate that within the range of parameter values derived from our experiments, the change in both individual and collective proportion of time spent scanning, as group size increases, depends only on the global mimetic propensity (ε_s_+ε_F_) and on the ratio between intrinsic foraging and scanning bouts durations (k_F_/k_S_). This makes the system remarkably robust in respect to parameter values that modulate both the individual propensity to imitate conspecifics (respectively, ε_s_ and ε_F_) and the intrinsic bout durations for foraging and scanning (respectively, 1/k_F_ and 1/k_S_).

**Figure 3:**
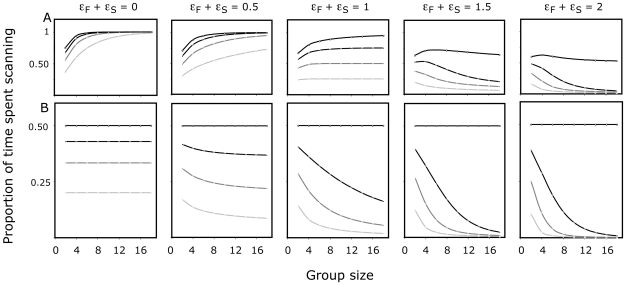
Model properties: the key individual decision-making factors modulating ‘the group size effect’. Effects of both the global mimetic propensity (ε_s_+ε_F_) and the differences between foraging and scanning bout durations (k_F_/k_S_) on the collective and the individual proportion of time spent scanning (A and B respectively) as a function of group size. The ratio k_F_/k_S_ equals to 1, 0.75, 0.5 and 0.25 for the open circles, the black dotted, the dark grey and light grey lines respectively (see Eq. (5) and (6)).

Figure 3 shows that depending on the sensitivity of start scanning or foraging to social influences, a great diversity of both individual and collective vigilance patterns may emerge as group size increases. When the decisions of individuals are independent of the activity of conspecifics (ε_s_ = ε_F_ = 0), group size does not affect the proportion of time an individual spends scanning (see Supporting information S3, Eq. (S.10c)). As sensitivity toward the activity of conspecifics increases, individual vigilance times decrease with group size. For ε_s_+ε_F_ = 1, the ratio between intrinsic foraging and scanning bouts durations shapes the group size effect (see also Supporting information S3, Eq. (S.11.c)). For ε_s_+ε_F_≥1 even weak differences between the bout durations of foraging and scanning are sufficient to drive the group size effect, but, at the same time, the level of collective vigilance decreases in large groups. Remarkably, such a result is in agreement with the diversity of the patterns of collective vigilance reported in the literature. It highlights the fact that for some values of k and ε, group size has a positive effect on the individual time allocated to foraging without hampering the efficiency of vigilance at the group level.

**Figure 4:**
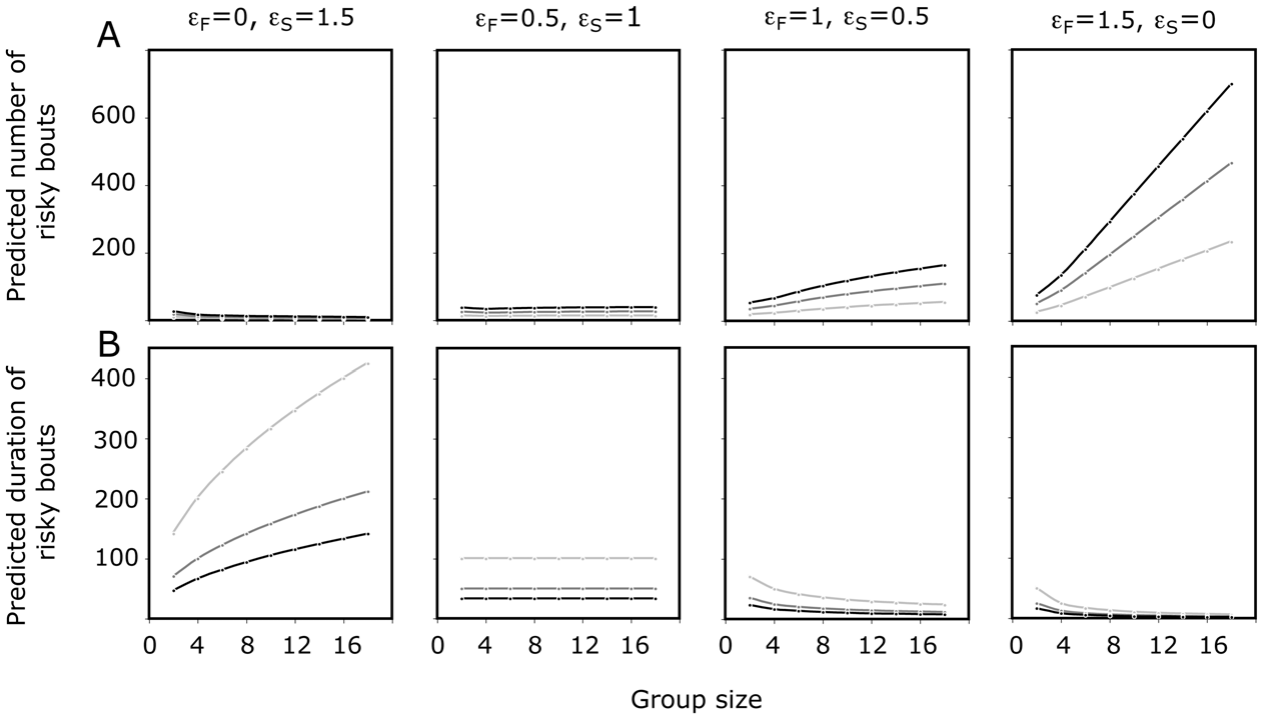
Model properties: the diversity of risk dynamics under constant ‘group size effect’. Fig. 4 shows the predicted dynamical features of risk as a function of group size, tempo and the propensity to imitate neighbors for either foraging or scanning decisions (respectively ε_F_ and ε_s_, see Eq. (1)). Figure 4A and 4B represent the predicted number and the average duration of bouts where no individual is scanning (F_0_ and <T_0_> respectively, see eq. (S.12d,e)), over a period of 4 hours when keeping constant both the global propensity to imitate conspecifics (ε_s_+ε_F_ = 1.5) and the ratio k_F_/k_S_ (equals to 0.5). Black, dark and light grey lines represent groups with high (k_F_ = 0.03, k_S_ = 0.06), medium (k_F_ = 0.02, k_S_ = 0.04) and low (k_F_ = 0.01, k_S_ = 0.02) tempo, respectively.

Within the range of the parameter values measured in our experiments with sheep, (i.e. ε_s_+ε_F_ = 1.5 and k_F_/k_S_ = 0.5), we further investigated the extent to which values of ε_s_, ε_F_, k_F_ and k_S_ affect the dynamical features of ‘risky’ situations, where no individual in the group is scanning (Figure 4, see Supporting information S2, Eq. (S.8) and (S.9)). Clearly, strong social influences on the individual decision to start scanning can lead to infrequent, but rather long bouts in risky situations (‘risky bouts’). In contrast, strong social influences on the individual decision to start foraging make the risky bouts more frequent and shorter as group size increases (Fig. 4). These results also highlight that for a constant global propensity to imitate conspecifics (ε_F_+ε_S_), variations in the respective weight of social influence in the decision to start either foraging or scanning (ε_F_ and ε_S_ respectively) lead to opposite strategies as group size increases although groups of the same size incur the same overall risk (see Fig. 4). Similarly, in all cases, while the overall risk remains the same, the individual tempo also modulates the dynamical features of collective vigilance, with less frequent but longer risky bout durations for groups with low tempo than for groups with high tempo (Fig. 4). Individual variation in the tempo, or rate of transition between activities, could therefore explain variability at the group level and lead to flexibility in behavior at the population level.

## Discussion

Our experimental and theoretical results elucidate the behavioral processes underlying the effect of group size on the dynamics of vigilance at both the individual and collective levels. Basically, a nonlinear response to the behavior of conspecifics modulates the probability of an individual switching activity (see fig. 1A,B). With the model, we show that such nonlinearities arise both from imitative processes (which are modulated by the parameter ε), and from the sensitivity of the individual response toward the ratio between scanning and foraging conspecifics (*N_S_* and *N_F_*, respectively). Such a competition between positive feedback loops for both scanning and foraging behavior constitutes a special feature of our model and results in the social amplification of even very small intrinsic differences in activity bout durations at an individual level (i.e. the theoretical behaviour of an isolated individual and its corresponding parameter values: k_F_ and k_S_), but at the same time maintains an efficient level of vigilance for the group. This contrasts with early assumptions that individual vigilance will decline as group size increases, either through explicit cognitive processes (the ‘many eyes effect’, [2]), or a reduced perception of danger due to a ‘dilution effect’ [31]. From a theoretical point of view, both of these assumptions would have resulted in a decrease of the propensity to spontaneously stop foraging activity (k_F_ parameter value) as group size increases, but this was not supported by our analyses (See Fig. 1C). Rather, our study confirms previous results suggesting that social facilitation acts as a key mechanism in the group size effect [15], [6], [32]–[33]. Our experiment and the possibility to test groups of various sizes under controlled conditions allowed formulating a model where parameter values were measured experimentally. Since both individual and collective patterns measured in merino sheep do not qualitatively nor quantitatively differ from those of their wild counterparts, we assume that the variations of the group size effect rely on the differences between parameter values across animal species rather than on different decision-making rules. Within the range of parameter values derived from our experiments, the model shows emergent properties whereby the decline of individual vigilance with increasing group size depends on only two key factors: 1) the global propensity to imitate conspecifics (i.e the sum of mimetic coefficients for both scanning and foraging decisions: ε_s_+ε_F_) and 2) the ratio between intrinsic foraging and scanning bout durations (k_F_/k_S_). With the model, we further explored collective properties when systematically varying the parameter values that modulate the social facilitation of both scanning and foraging decisions and monitoring its effect on time allocation patterns and collective vigilance dynamics. Our results show a wide range of collective and individual vigilance patterns, from which it is possible to identify a small number that are consistent with empirical studies and which result from a small number of combinations of parameter values. Typically, the model predicts that individual foraging should increase while individual vigilance decreases as group size increases as soon as animals show a propensity to imitate conspecifics (for ε_s_+ε_F_>0 see Fig. 3) and that an intrinsic difference between scanning and foraging bout durations exists. On the other hand, we show that collective vigilance is subjected to a great diversity of patterns depending on the magnitude of differences between intrinsic bouts durations and on the strength of the global propensity to imitate conspecifics. For example, collective vigilance increases when the propensity to imitate conspecifics is moderate (ε_s_+ε_F_<1), but decreases or increases up to a pivotal group size and then decreases when the propensity to imitate conspecifics becomes high (for ε_s_+ε_F_>1 see Fig. 3). In such cases, large group formation might still be beneficial for individuals because they also benefit from other factors, unrelated to collective vigilance, like for instance the dilution effect. Further studies are needed to understand the relative benefits of the dilution effect and of the decrease in collective vigilance.

An other result of our modeling approach leads to the conclusion that the ‘group size effect’ is extremely robust in respect to variations in the parameter values that modulate the propensity to imitate and the duration of both scanning and foraging activity. For example, for a constant global propensity to imitate conspecifics (ε_F_+ε_S_), groups of the same size incur the same overall risk. Similarly, for a constant ratio between intrinsic foraging and scanning bout durations (k_F_/k_S_), groups of the same size that show variations in ‘tempo’, incur the same overall risk (P(0)). However, when investigating the dynamical features of risky situations, we show that collective vigilance is strongly influenced by the absolute parameter values that modulate the respective propensity to imitate conspecifics in foraging and scanning decisions (ε_F_ and ε_S_) and the activity transition rates (k_F_ and k_S_). For example variations in the respective weight of social influence for making the decision to start either foraging or scanning (ε_F_ and ε_S_ respectively) lead to opposite strategies at the level of group vigilance (alternatively, frequent and short risky bouts or rather rare but long risky bouts) with opposite tendencies as group size increases (see Fig. 4). Similarly for groups with the same value for the ratio k_F_/k_S_, variations in the tempo have dramatic consequences for the dynamics of risky situations. These results highlight the fact that small variations in individual vigilance can lead to flexibility in the dynamical properties of collective vigilance and should encourage future studies about vigilance to further explore the dynamic properties of collective vigilance under natural conditions.

Since each situation is characterized by different temporal pattern of collective vigilance, it is likely that both predation pressure and perceptual capability played a crucial role in the evolution of the individual propensity to imitate conspecifics. Then a challenging question is to what extent animal species and populations that were exposed to varying predation or foraging pressures differ in their propensity to imitate and in their intrinsic probability of changing activity. Measuring the form of such responses across species, and their link to evolutionary pressures, will help determine the importance of mimetic forces in the evolution of group living. Basically, the identification of the key processes involved in the group size effect produces a theoretical framework in which it is possible to understand how evolutionary pressures might have shaped group vigilance patterns.

Several sources of variation in the key behavioral parameters might affect the dynamics of vigilance. For instance the biological constraints that may contribute to variations in scanning and foraging bout durations are diverse (i.e. perceptual capability, food handling constraints, motivation) and are likely to vary both across animal species and within and between individuals of the same species [34]–[35]. Additionally it is likely that the position of an animal within the group has an effect on its psycho-physiological state [36]–[39], which might affect its spontaneous propensity to stop foraging and/or scanning (k parameter values). Moreover, the intriguing relationship between the intrinsic bouts durations of foraging and scanning (k_F_ and k_S_, see Fig. 1C) suggests the existence of groups with high and low tempo. Such variations probably arise from differences between the individuals in any group and thus would be consistent with inter-individual differences in activity level and the concept of behavioral profiles broadly reported in the literature [40]. As a consequence, in the case of conspecifics differing in tempo, this relationship would limit the range of differences in activity budgets and their related energy expenditure [41]. Because these intrinsic tempos also clearly affect the risk of failing to detect a predator, determining how individual characteristics modulate the spontaneous propensity to switch activity (i.e. k parameter values) remains an exciting theoretical and experimental challenge. Further research is needed to clarify the sources of variation within and between individuals, and to show how individual differences are integrated at the level of the group.

From a functional standpoint, socially facilitated scanning behavior is likely to increase individual fitness, since any benefits of collective vigilance depend on the rapid transmission of information about the approach of a predator from those individuals that are vigilant to those that are not [33], [42]–[44]. On the other hand, socially facilitated feeding behaviors and the use of public information for making foraging decisions have stimulated extensive research efforts, which have acknowledged the effect on individual benefits [45]–[48]. Our study provides new insights into the processes whereby information about the activity of conspecifics is used in decisions to switch between scanning and foraging activities at an individual level. In the model, the lower the mimetic propensity (value of parameter ε), the less the decision-making depends upon conspecific activity and the more it depends on personal information. Our results for sheep place significant weight on ‘public information’ when deciding to start scanning whereas the decision to start foraging relies to a greater extent on ‘personal information’ (ε_s_>ε_F_). More generally, we demonstrate how function and parameters governing social interactions, and thereby collective dynamics, are linked to the personal and public information and their use by animals. We assume that individuals are able to discriminate between different behavioral patterns exhibited by the conspecifics. As a consequence, our results lead us to rethink the relationship between individual capabilities, the integration of information and the collective response. There are many studies concerned with collective decision-making in invertebrates and vertebrates, which focus on imitative behavior [49]. Most of them hypothesize that the individual probability of switching activity depends only on the number of individuals engaged in one type of behavior. Our assumption is that, in most of the cases, the probability of switching behavior depends on a combination of the number of individuals in different behavioral states. For example, in social insects, during the nest-moving or the foraging, it can be the number of both recruiting and aggregated insects that is important [12], [50] while a study of sheep by Gautrais et al [24] showed that in transitions from activity to inactivity, decisions are modulated by both active and resting conspecifics. As a consequence, as the number of variables governing the decision-making increases (i.e. in the case when several different behaviours are considered) the number of feedback loops involved in the social network and therefore the diversity of collective response increases. Another consequence arises from the relationship between collective responses and group size. Since in our model the probability of switching activity depends on the ratio between numbers of individuals engaged in each behavior, the sensitivity of the collective response to group size may be very different from that in systems where the absolute number of individuals is important, such as the decision to move in fish [51]–[52] and in primates [53]–[55]. In contrast with studies emphasizing the simplicity of individual behavior over the collective complexity of the task being performed (e.g. Swarm intelligence, [56]), our work is a first step towards reconsidering this paradigm and learning how the intrinsic capabilities of individual to process information affect the complexity of group behavior and how collective information processing, in turns, affect individual fitness.

This study provides evidence that the adaptive individual response to the trade-off between feeding and vigilance in social animals emerges from a combination of intrinsic differences of activity bout durations and imitative behavior. The robustness and flexibility of the system suggest a generic aspect to this principle. The sensitivity of the system to tempo, at an individual level, emphasizes the necessity for integrating both social facilitation effects and idiosyncratic responses into quantitative models of time allocation patterns and collective vigilance dynamics. Our assumption about activity discrimination raises the question of the links between individual capability and the complexity of collective responses. We thus provide further insight into the links between functional explanations of the evolution of decision rules in animal groups and their proximate explanations.

## Supporting Information

Supporting Information S1 Expected probability of scanning and foraging number of individuals.
(DOC)

Supporting Information S2 Model properties within the range of parameter values experimentally estimated.
(DOC)

Supporting Information S3 Model sensitivity towards imitation and mathematical simplifications.
(DOC)
