# Supplementary material for: How Group Size Affects Vigilance Dynamics and Time Allocation Patterns: The Key Role of Imitation and Tempo
Source: PLoS One. 2011 Apr 15;6(4):e18631. doi: 10.1371/journal.pone.0018631 (PMC3078120; doi:10.1371/journal.pone.0018631)
Supplement: Supporting Information S1 — Expected probability of scanning and foraging number of individuals. (DOC) [file pone.0018631.s001.doc]

**Supporting information: Text 1**

*Expected Probability of scanning and foraging number of individuals*

The master equation, a set of first-order differential equations, describes the time evolution of the probability of the system to occupy each one of the discrete sets of states. *P(N_S_)* is the probability for the system to be in state *N_S_*. *N_S_* are the number of scanning individuals (*N_S_* =*0,1,….,N*). N is the total number of individuals and the number of foraging individuals *N_F_* =*N*-*N_S_*. The equation counts the processes leading the system to the sate *N_S_* and the processes removing it from this state:

where *V^+^* is the contribution of transitions to state *N_S_* per unit time and *V^-^* is the loss from state *N_S_* to other states of the system per unit time.

V^+^ is the product of transition probability per unit time of going from state *N_S_* +1 to *Ns*, multiplied by the probability of being in the state *N_S_* +1 (P(*N_S_+1))* at time *t* plus the product of transition probability per unit time of going from state *N_S_* -1 to *N_S_*, times the probability of being in the state *N_S_* -1 (P(*N_S_* -1*))* at time *t*.

The two transitions leading to the state (*Ns*) are (see Flowchart S1):

*N_S_+1, N_F_-1 → N_S_*, *N_F_*: one of the *N_S_ +1* scanning sheep starts to forage.

*N_S_ -1, N_F_ +1 , → N_S_, N_F_*: one of the *N_F_+1* foraging sheep starts to scan.

To each term, it corresponds a transition probability, equal to the individual probability of starting a new behaviour times the number of individuals able to perform the corresponding behavior. For example, the transition probability (*T’*) between (*Ns+1*) and (*Ns)* is equal to the individual probability per unit of time of starting to forage multiplied by the number of individuals scanning (*Ns+1*).

Similarly, *V^-^* is the product of the probability of being in state *N_S_* at time *t*, multiplied by the sum of the transition probabilities per unit time from *N_S_* to all other states (*N_S_* +1 , *N_S_* -1 ) accessible from *N_S_*.

The two terms contributing to the decrease of *N_S_*:

*N_S_, N_F_-1 → N_S_* -1,*N_F_*: one of the *N_F_* sheep starts to forage.

*N_S_, N_F_ , → N_S_+1, N_F_*-1: one of the *N_F_+1* sheep starts to scan.

At the steady state the probability of N_S_ individuals scanning within a group of N sheep was computed as follow:

**Flowchart S1:** Flowchart illustrating the transition probabilities between the different states of the system (birth terms: black arrows, death terms: black dotted arrows). *Ns* and *N_F_* represent the number of individual scanning and foraging respectively.

Based on our experimental results, the master equations were fitted with the function:

These results allow determining the probability of each state of the system, which in our case corresponds to each possible number of individual scanning in the groups:

and

This result also allows determining the proportion of time where at least one individual is scanning (collective vigilance P_Coll_) as follow:

with

(S.5)
