# Supplementary material for: How Group Size Affects Vigilance Dynamics and Time Allocation Patterns: The Key Role of Imitation and Tempo
Source: PLoS One. 2011 Apr 15;6(4):e18631. doi: 10.1371/journal.pone.0018631 (PMC3078120; doi:10.1371/journal.pone.0018631)
Supplement: Supporting Information S2 — Model properties within the range of parameter values experimentally estimated. (DOC) [file pone.0018631.s002.doc]

**Supporting information: Text 2**

*Model properties within the range of parameter values experimentally estimated*

For the parameters values experimentally estimated, with A= 1, the equation *S.5* becomes:

and *S.3a* becomes

It is easy to show that

and therefore S.6b becomes:

Thus the probability of risky situation, where no individual is scanning is:

The frequency F_0_ of the occurrence of a risky situation was given by:

(S.8)

The calculated probabilities were assimilated to the events frequencies by second. As a consequence these probabilities were multiplied by 14 400 in order to make direct comparison to the average amount of time of one observation period.

The average duration <T_0_> of a risky bout is given by:
