# Supplementary material for: How Group Size Affects Vigilance Dynamics and Time Allocation Patterns: The Key Role of Imitation and Tempo
Source: PLoS One. 2011 Apr 15;6(4):e18631. doi: 10.1371/journal.pone.0018631 (PMC3078120; doi:10.1371/journal.pone.0018631)
Supplement: Supporting Information S3 — Model sensitivity towards imitation and mathematical simplifications. (DOC) [file pone.0018631.s003.doc]

**Supporting information: Text 3**

*Model sensitivity towards imitation and mathematical simplifications:*

Theoretical predictions when individual decisions are independent of the activity of conspecifics, for the ε_S_ = ε_F_ = 0:

Theoretical predictions for ε_S_ + ε_F_ = 1
